# Informatics can help providers incorporate context into care

**DOI:** 10.1093/jamiaopen/ooy025

**Published:** 2018-06-22

**Authors:** Hossein Estiri, Chirag J Patel, Shawn N Murphy

**Affiliations:** 1 Harvard Medical School, Boston, Massachusetts, USA; 2 Laboratory of Computer Science, Department of Medicine, Massachusetts General Hospital, Boston, Massachusetts, USA; 3 Partners Healthcare, Boston, Massachusetts, USA; 4 Department of Biomedical Informatics, Boston, Massachusetts, USAand; 5 Department of Neurology, Massachusetts General Hospital, Boston, Massachusetts, USA

**Keywords:** Social Determinants of Health, informatics, EHR

## Abstract

Most determinants of health originate from the “contexts” in which we live, which has remained outside the confines of the U.S. healthcare system. This issue has left providers unprepared to operate with an ample understanding of the challenges patients may face beyond their purview. The recent shift to value-based care and increasing prevalence of Electronic Health Record (EHR) systems provide opportunities to incorporate upstream contextual factors into care. We discuss that incorporating context into care is hindered by a chicken-and-egg dilemma – ie, lack of evidence on the utility of contextual data at the point of care, where contextual data are missing due to the lack of an informatics infrastructure. We argue that if we build the informatics infrastructure today, EHRs can give the tomorrow’s clinicians the tools and the data they need to transform the U.S. healthcare from episodic and reactive to preventive and proactive. We also discuss system design considerations to improve efficacy of the suggested informatics infrastructure, which include systematically prioritizing contextual data domains, developing interoperability standards, and ensuring that integration of contextual data does not disrupt clinicians’ workflow.

## INTRODUCTION

Despite mounting expenditure on healthcare, the U.S. lags behind many industrial nations in several health indicators and has glaring health disparities.^1–4^ Part of the poor returns from the inordinate amount of dollars invested in the healthcare system has been attributed to an endemic overemphasis on clinical care.^5^

Over the past 4 decades, emerging data has suggested that many determinants of our health originate from the contexts in which we live.^6^ In social epidemiology, many of these factors are often studied under the rubric of social determinants of health. The notion of “context” originates from an ecological perspective to health that describes an individual’s health status as the result of interactions between multiple levels of factors, including biological, psychological, social, cultural, historical, institutional, political, and environmental factors.^7^^,^^8^ Context is a reflection of the place (exposures and proximities), institutions, and actors (ie, the people), and the complex interactions between them,^9^ as they determine community and individual health outcomes. The contextual factors along with lifestyle (which is indirectly shaped by the context), determine our health more than our genetics do.^10^ Today, these factors are usually outside the confines of the U.S. healthcare system,^6^^,^^11–18^ and healthcare providers are unprepared to operate with an ample understanding of the challenges patients face outside the providers self-imposed domain.

## OPPORTUNITIES

But there is hope and informatics is a major means to it. The recent shift to value-based care and increasing prevalence of Electronic Health Record (EHR) systems have created an opportunity to transition the healthcare system from a 1-size-fits-all medicine to enable health services that incorporates contextual factors upstream.

Since the early 2000s, several national advisory committees have made explicit recommendations, promoting inclusion of an array of non-clinical determinants of health to maximize the benefits of national investments on EHRs and health information exchange.^19–21^ Most particularly, in 2014, the Institute of Medicine published 2 reports to elaborate the need and required actions to augment the EHR systems with data on contextual domains, such as education, indicators of financial resource strain, social connections and isolation, exposure to violence, and neighborhood and community compositional characteristics.^6^^,^^14^ There are technical solutions to this data integration problem. However, augmenting the EHR to collect and encapsulate these data poses a number of challenges that need to be addressed.

## AUGMENTING THE EHR DATA MODEL: SOLUTIONS AND CHALLENGES

**Figure 1.**
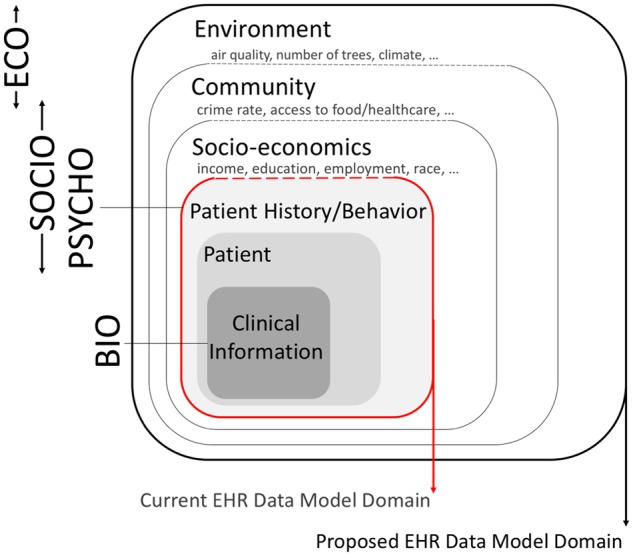
Data model domains represented in an augmented EHR—Adapted from Ref.^22^

An expansion beyond the traditional clinical information collected EHR systems would require augmenting the present EHR data models from representing merely biological to bio-psycho-socio-eco repositories (Figure 1).

A few institutions^13^^,^^23–26^ have already begun investigating ways to integrate contextual data into EHRs. Two solutions have been applied to this task. The first one is collecting individual-level contextual data directly from patients through surveys that can be incorporated at the point of care—for example, see Gold et al.^27^ Patient-reported solutions can collect individual-level information such as income and education, however, may fall short in collecting contextual information, such as the socioeconomic characteristics of the neighborhood where a patient resides or receives healthcare. Also, some of these individual-level information (eg, poverty status, income) may be perceived as sensitive or irrelevant by patients, adding to the complexities of collecting reliable information directly from patients without creating uncomfortable situations. A second approach is using geospatial/temporal technology to join group-level information to individual patient records—for example, see Bazemore et al. and Hughes et al.^13^^,^^23^ The increasing availability of data from social media, urban sensors, and agencies such as the Census Bureau, coupled with the available technological resources makes the group-level data collection-and-integration solution viable. However, making individual-level interpretations from aggregated group-level data will be challenging—this challenge is known as ecological fallacy.^28–30^ Further, some contextual information (eg, access to healthy food or clinical care), need to be computed dynamically, introducing further computational challenges.

Data sharing, governance, and privacy concerns introduce additional challenges that discourage healthcare organizations from integrating non-clinical data into their clinical repositories.^13^^,^^25^^,^^31–33^ Moreover, clinicians already struggle to incorporate the mounting loads of information into treatment choices for patients.^34^ Without robust evidence of clinical utility, it will be difficult to persuade healthcare organizations to add new layers of non-clinical data to their repositories.

Available technologies (eg, geospatial data integration software solutions, Fast Healthcare Interoperability Resources, SMART Health IT) can help with collecting contextual data and presenting them in EHRs. In addition, new and emerging technologies, such as wearable devices or food consumption trackers, can yield individual-level behavioral and contextual data and push them to EHR repositories. Regardless of how data are collected, though, there will be new cultural challenges. How can we incent patients to disclose their, for example, socioeconomic information, whether it be collected directly or indirectly, before clearly demonstrating how contextual information can be directly applied to improving their health?

## ENVISIONING CLINICAL UTILITY OF DATA FROM CONTEXT

Despite the available epidemiological knowledge on a few population health interventions based on contextual factors, there is little known about best methods to intervene on contextual data in clinical settings. We still lack enough empirical evidence to support whether contextual data can be directly incorporated in clinical interventions^12^ to improve precision in diagnosis, treatment, and monitoring of disease.

There are multiple possible scenarios where adding contextual data to clinical data already store in EHRs could improve patients’ health. Adding contextual covariates to prognostic models could improve the amount of variance in disease risk explained by the models. An example would be adding patients’ income level and access to fast foods could improve our ability to predict risk of obesity. In this case, a decision support system can produce a more precise obesity risk factor for the care provider to take action. Adding contextual data could also improve our current knowledge about the role of known clinical (and genetic) determinants of a given health outcome. For example, body mass index and hypertension are known risk factors in most diabetes risk models. Adding contextual determinants, such as access to healthy food, may complement existing models in estimating risk of diabetes.

The augmented EHR system would facilitate shared decision making between the patients and providers for establishing personalized health goals that consider availability of resources. For example, non-adherence to prescribed medications is often the result of the inability to afford the medications, but this is often hidden by the patient to the provider due to shame. Economic indicators could bring this to light during an encounter. The augmented EHR system may also enable healthcare organizations/practices to improve precision in diagnosis, treatment, and monitoring of disease and care management programs, tailor their services, prioritize their resource, and identify hot spots of disease prevalence and at- risk populations. For example, accounting for contextual information can result in a more precise cholesterol treatment, compared with the conventional, purely medical procedures, such as the Framingham risk score factors.^35^ Demonstrating that at least one of these possible scenarios are feasible will support the clinical utility of contextual information.

## INFORMATICS IS THE MISSING LINK

Despite the seemingly evident knowledge and resounding endorsements on the importance of contextual factors in determining individual and population health, a scalable informatics infrastructure for incorporating context into clinical care is yet to be developed. This, in our opinion, is partially due to the fact that there is not enough evidence to support the clinical utility of this information.

**Figure 2.**
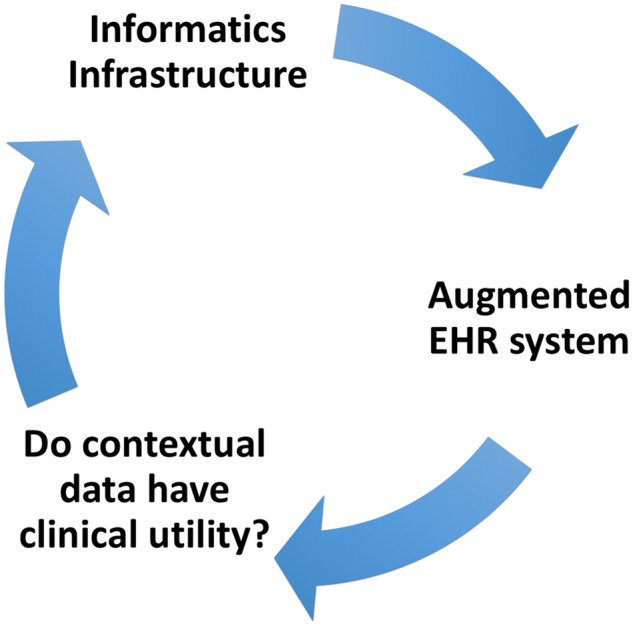
A chicken-and-egg dilemma formed from interdependencies between integrated data, informatics infrastructure, and clinical utility of contextual data.

For testing the clinical utility of contextual data, we need these data integrated with the clinical data in an augmented EHR system, which, in turn, is dependent on availability of an informatics infrastructure to integrate those data with EHRs. To support development of an informatics infrastructure, clinical utility of contextual data needs to be proven. These interdependencies form a chicken-and-egg dilemma that has yet to be sorted out (Figure 2).

To sort this dilemma out, we need to start developing an adept informatics infrastructure that leverages available technologies to collect and assimilate contextual data within EHRs and has the capacity to support clinical workflow management and decision support systems. If we build the informatics infrastructure today, EHRs can give tomorrow’s clinicians the tools and the data they need to transform the U.S. healthcare from an episodic reactive model to a preventive and proactive system.

## DISCUSSION

Certain system design considerations can improve the efficacy of the informatics infrastructure towards incorporating context into care, including: (1) prioritization of contextual data domains, (2) the necessity for interoperability standards, and (3) smooth integration of contextual data with clinicians’ workflow.

Currently, we lack a systematic strategy to identify, prioritize, and address contextual data in clinical settings.^21^ The 2014 IOM reports recommended the inclusion of data on education, financial resource strain, social connections and isolation, exposure to violence, and neighborhood and community compositional characteristics into EHRs.^6^^,^^14^ These data domains are broad. Additionally, individual determinants of a health outcome under each domain may vary in effect and importance. Contextual data domains need to be prioritized based on evidence/possibility of known clinical or policy interventions, availability and granularity of contextual data, contextual relevance, and local and state needs.^12^^,^^21^^,^^31^

In addition, current standards are insufficient to ensure interoperability of EHR with other technologies which can collect and present data about patients’ context. Contextual information needed for healthcare decision-making reside in disparate sources and administrative structures.^21^ Without interoperability standards, efforts to integrate contextual data with EHRs across different informatics platforms will remain cluttered. Moreover, high throughput of non-clinical data from disparate sources can cause performance issues in clinical repositories. It is likely that solutions that link fluid registries of information to high performance databases, like the Informatics for Integrating Biology and the Bedside (i2b2),^36^ will be needed to integrate the copious information into sizable chunks.

Because work on applying Health Information Technology to address incorporation of context into care is still in its infancy, many of the recommendations on the necessity for integrating contextual data with EHRs (specifically, the IOM reports^6^^,^^14^) are based on an implicit assumption that, if these data are captured in EHRs, we will be able to somehow incorporate them in clinical care. However, simply documenting contextual data in EHR systems will not be sufficient for clinical decision making at the point of care.^2^^,^^13^^,^^21^^,^^37^^,^^38^ Once we have the data in EHRs, we would also need to present and integrate the new data into clinical workflows and develop decision support systems that would harness contextual data to prompt action.

Finally, as the informatics infrastructure will involve integrating individual-level clinical data with aggregate-level contextual data from various non-clinical sources, potential issues related to data sharing, governance, ethics, medical paternalism, and ecological fallacy (when making inference from the integrated data) need to be acknowledged and address adequately.

*Conflict of interest statement*. None declared.

## References

[1] American Academy of Nursing. Putting “health” in the electronic health record: a call for collective action. Nurs Outlook 2015; 63 (5): 614–6. 26598771 10.1016/j.outlook.2015.08.003

[2] DeVoe JE, Bazemore AW, Cottrell EK, et al Perspectives in primary care: a conceptual framework and path for integrating social determinants of health into primary care practice. Ann Fam Med 2016; 14 (2): 104–8. 26951584 10.1370/afm.1903 PMC4781512

[3] McGovern L, Miller G, Hughes-Cromwick P. Health policy brief: the relative contribution of multiple determinants to health outcomes. Health Aff 2014 http://www.rwjf.org/content/dam/farm/reports/issue_briefs/2014/rwjf415185 (Accessed February 1, 2016).

[4] OECD. Health at a Glance 2015: OECD Indicators. Paris: Organisation for Economic Co-operation and Development; 2015 doi: 10.1787/health_glance-2015-en.

[5] Schroeder SA. We can do better—improving the health of the American people. N Engl J Med 2007; 357 (12): 1221–8. 17881753 10.1056/NEJMsa073350

[6] Institute of Medicine. Capturing Social and Behavioral Domains and Measures in Electronic Health Records: Phase 2. Washington, DC: The National Academies Press; 2014. 25590118

[7] Sallis JF, Owen N, Fisher EB. Ecological Models of Health Behavior. 2008. doi: 10.7326/0003-4819-116-4-350_1.

[8] Green LW, Richard L, Potvin L. Ecological foundations of health promotion. Am J Health Promot 1996; 10 (4): 270–81. 10159708 10.4278/0890-1171-10.4.270

[9] Frohlich KL, Corin E, Potvin L. A theoretical proposal for the relationship between context and disease. Sociol Health Illness 2001; 23 (6): 776–97.

[10] Amador C, Xia C, Nagy R, et al Regional variation in health is predominantly driven by lifestyle rather than genetics. Nat Commun 2017; 8 (1): 801. 28986520 10.1038/s41467-017-00497-5 PMC5630587

[11] Busza J, Walker D, Hairston A, et al Community-based approaches for prevention of mother to child transmission in resource-poor settings: a social ecological review. J Int AIDS Soc 2012; 15 (Supp 2): 17373. 22789640 10.7448/IAS.15.4.17373 PMC3499910

[12] Glasgow RE, Kaplan RM, Ockene JK, Fisher EB, Emmons KM. Patient-reported measures of psychosocial issues and health behavior should be added to electronic health records. Health Aff (Millwood) 2012; 31 (3): 497–504. 22392660 10.1377/hlthaff.2010.1295

[13] Bazemore AW, Cottrell EK, Gold R, et al “ Community Vital Signs”: incorporating geocoded social determinants into electronic records to promote patient and population health. J Am Med Inform Assoc 2015; doi: 10.1093/jamia/ocv088. 10.1093/jamia/ocv088 PMC11740537 26174867

[14] Institute of Medicine. Capturing Social and Behavioral Domains in Electronic Health Records: Phase 1. Washington, DC: National Academies Press; 2014. 24757748

[15] Braveman P, Gottlieb L. The social determinants of health: it’s time to consider the causes of the causes. Public Health Rep 2014; 129 (Suppl 2): 19–31. 10.1177/00333549141291S206 PMC3863696 24385661

[16] McGinnis JM, Williams-Russo P, Knickman JR. The case for more active policy attention to health promotion. Health Aff 2002; 21 (2): 78–93. 10.1377/hlthaff.21.2.78 11900188

[17] Pantell M, Rehkopf D, Jutte D, Syme SL, Balmes J, Adler N. Social isolation: a predictor of mortality comparable to traditional clinical risk factors. Am J Public Health 2013; 103 (11): 2056–62. 24028260 10.2105/AJPH.2013.301261 PMC3871270

[18] Geronimus AT, Bound J, Ro A. Residential mobility across local areas in the United States and the geographic distribution of the healthy population. Demography 2014; 51 (3): 777–809. 24781651 10.1007/s13524-014-0299-4 PMC4129513

[19] Smedley BD, Syme SL. Promoting health: intervention strategies from social and behavioral research. Am J Health Promot 2001; 15 (3): 149–66. 11265579 10.4278/0890-1171-15.3.149

[20] NCVHS. Toward Enhanced Information Capacities for Health: An NCVHS Concept Paper. Washington, DC; 2010 http://www.ncvhs.hhs.gov/wp-content/uploads/2014/05/100526concept.pdf (Accessed February 1, 2016).

[21] Institute of Medicine. For the Public’s Health: The Role of Measurement in Action and Accountability. Washington, DC: The National Academies Press; 2011. 24983050

[22] Dahlgren G, Whitehead M. Policies and strategies to promote social equity in health. Stock Inst Futur Stud 1991; doi: 978-91-85619-18-4.

[23] Hughes LS, Phillips RLJ, DeVoe JE, Bazemore AW. Community vital signs: taking the pulse of the community while caring for patients. J Am Board Fam Med 2016; 29 (3): 419–22. 27170802 10.3122/jabfm.2016.03.150172

[24] Roth C, Shivade CP, Foraker RE, Embi PJ. Integrating population- and patient-level data for secondary use of electronic health records to study overweight and obesity. Stud Health Technol Inform 2013: 192: 1100. 23920874

[25] Bazemore A, Phillips RL, Miyoshi T. Harnessing Geographic Information Systems (GIS) to enable community-oriented primary care. J Am Board Fam Med 2010; 23 (1): 22–31. 20051539 10.3122/jabfm.2010.01.090097

[26] Simpson CL, Novak LL. Place Matters: the problems and possibilities of spatial data in electronic health records. AMIA Annu Symp Proc 2013; 2013: 1303–11. 24551409 PMC3900146

[27] Gold R, Cottrell E, Bunce A, et al Developing Electronic Health Record (EHR) Strategies Related to Health Center Patients’ Social Determinants of Health. J Am Board Fam Med 2017; 30 (4): 428–47. 28720625 10.3122/jabfm.2017.04.170046 PMC5618800

[28] Berkowitz SA, Traore CY, Singer DE, Atlas SJ. Evaluating area-based socioeconomic status indicators for monitoring disparities within health care systems: results from a primary care network. Health Serv Res 2015; 50 (2): 398–417. 25219917 10.1111/1475-6773.12229 PMC4369215

[29] Freedman DA. Ecological inference and the ecological fallacy. Int Encycl Soc Behav Sci 1999; 6 (4027–4030): 1–7.

[30] Robinson WS. Ecological correlations and the behavior of individuals. Am Sociol Rev 1950; 15 (3): 351–7. 10.1093/ije/dyn357 19179346

[31] Kukafka R, Ancker JS, Chan C, et al Redesigning electronic health record systems to support public health. J Biomed Inform 2007; 40 (4): 398–409. 17632039 10.1016/j.jbi.2007.07.001

[32] Mathys T, Kamel Boulos MN. Geospatial resources for supporting data standards, guidance and best practice in health informatics. BMC Res Notes 2011; 4 (1): 19. 21269487 10.1186/1756-0500-4-19 PMC3224535

[33] Comer KF, Grannis S, Dixon BE, Bodenhamer DJ, Wiehe SE. Incorporating geospatial capacity within clinical data systems to address social determinants of health. Public Health Rep 2011; 126 (Suppl 3): 54–61. 21836738 10.1177/00333549111260S310 PMC3150130

[34] Murdoch T, Detsky A. The inevitable application of big data to health care. J Am Med Inform Assoc 2013; 309 (13): 1351–2. 10.1001/jama.2013.393 23549579

[35] Franks P, Tancredi DJ, Winters P, Fiscella K. Including socioeconomic status in coronary heart disease risk estimation. Ann Fam Med 2010; 8 (5): 447–53. 20843887 10.1370/afm.1167 PMC2939421

[36] Murphy SN, Weber G, Mendis M, et al Serving the enterprise and beyond with informatics for integrating biology and the bedside (i2b2). J Am Med Inform Assoc 2010; 17 (2): 124–30. 20190053 10.1136/jamia.2009.000893 PMC3000779

[37] Williams DR, Costa MV, Odunlami AO, Mohammed SA. Moving upstream: how interventions that address the social determinants of health can improve health and reduce disparities. J Public Health Manag Pract 2008; 14 (Suppl): S8–17. 18843244 10.1097/01.PHH.0000338382.36695.42 PMC3431152

[38] Sadana R, Harper S. Data systems linking social determinants of health with health outcomes: advancing public goods to support research and evidence-based policy and programs. Public Health Rep 2011; 126 (Suppl 3): 6–13. 21836730 10.1177/00333549111260S302 PMC3150122

